# Enhanced Resistive Switching and Synaptic Characteristics of ALD Deposited AlN-Based RRAM by Positive Soft Breakdown Process

**DOI:** 10.3390/ijms232113249

**Published:** 2022-10-31

**Authors:** Seyeong Yang, Jongmin Park, Youngboo Cho, Yunseok Lee, Sungjun Kim

**Affiliations:** Division of Electronics and Electrical Engineering, Dongguk University, Seoul 04620, Korea

**Keywords:** RRAM, aluminum nitride, conduction mechanism, synaptic devices

## Abstract

Nitride film played an essential role as an excellent diffusion barrier in the semiconductor field for several decades. In addition, interest in next-generation memories induced researchers’ attention to nitride film as a new storage medium. A Pt/AlN/TaN device was investigated for resistive random-access memory (RRAM) application in this work. Resistive switching properties were examined in the AlN thin film formed by atomic layer deposition (ALD). The unique switching feature conducted under the positive voltage was investigated, while the typical bipolar switching was conducted under the application of negative voltage. Good retention and DC, and pulse endurances were achieved in both conditions and compared to the memory performances. Finally, the electronic behaviors based on the unique switching feature were analyzed through X-ray photoelectron spectroscopy (XPS) and the current–voltage (I–V) linear fitting model.

## 1. Introduction

The semiconductor field is expanding to meet the production of information, and the importance of memory function is increasing in line with the growth of the market [1]. Memory density needs to be increased constantly to meet the growing information society [2]. However, the challenge of scaling down in dynamic random-access memory (DRAM) and flash memory compels researchers to give attention to alternative memories, such as resistive RAM (RRAM), phase-change RAM(PRAM), and ferroelectric RAM (FRAM) [3,4,5]. Especially, RRAM with a simple structure satisfies the demand for high scalability, low-power operation, fast switching speed, and long retention [6,7]. Thus, it is suitable to replace traditional memory and has sufficient demand from memory application development.

Various metal oxides are studied over decades and are still being used as a storage medium in RRAM to store the information [8,9,10]. Numerous studies have been conducted; however, problems such as current overshoot and inconsistent forming voltage remain [11,12]. To solve those problems, the nitride compound semiconductors (e.g., BN, Si_3_N_4_, and AlN) are discovered and attracting many research groups as a new storage medium for RRAM. Good dielectric properties, high resistivity, and chemical compatibility with nitride electrodes are fundamentally included; in particular, AlN with a high energy bandgap has the advantage of an insulating layer in RRAM [13,14,15,16,17].

Sputtering and ALD are representative methods to deposit AlN film [18,19]. Both methods are sufficient to be used in RRAM process; however, the exact thickness control of ALD in the nanometer unit provides excellent resistance state reproducibility. In addition, since the RRAM switching is affected by the number of defects and impurities, ALD with perfect uniformity, superior film quality, and step coverage provides an inevitable choice [20]. Thus, we deposited a 5 nm AlN thin film on the TaN electrode using ALD, and formed an electrode with the inert metal of Pt for RRAM fabrication.

In this work, we investigated the resistive and synaptic properties of Pt/AlN/TaN devices. Resistive switching characteristics were identified by controlling the bias voltage. Unique switching behaviors and positive soft breakdown (PSB) were observed in the different voltage conditions from the bipolar switching. The memory performances, such as high resistance state (HRS), endurance, and retention were affected by these conditions. It is found that stable switching properties could be achieved after the PSB. The filamentary switching model and I–V linear fitting were used to analyze the influence of the soft breakdown on resistive switching.

## 2. Results and Discussion

**Figure 1:**
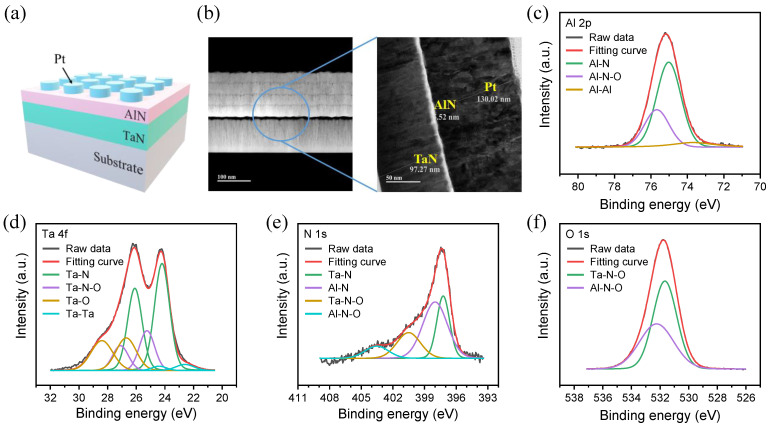
(**a**) Schematic of structure and components of Pt/AlN/TaN device. (**b**) TEM image. XPS spectra of (**c**) Al 2p, (**d**) Ta 4f, (**e**) N 1s, and (**f**) O 1s scan AlN/TaN within 10 nm.

The schematic of the Pt/AlN/TaN device is shown in Figure 1a, and the thickness of each layer was confirmed by transmission electron microscopy (TEM) images in Figure 1b. A 5.52 nm thick AlN layer was measured in a TEM image. The surface of AlN film, deposited on the TaN layer, was analyzed by XPS to detect the element spectra of Al, N, O, and Ta. Al-N bonding was at ~75 eV in the middle of Al-N-O bonding. Al-Al bonding shows a strong intensity in Figure 1c. The ratio between Al-N bonding and Al-N-O bonding is around 2.25:1, indicating that the oxygen was incorporated in the AlN film. Oxidation could be progressed on the TaN and AlN surfaces in the air, and some oxygen may remain in the chamber during AlN deposition. It is noted that most of the existing AlN film was also oxidized [21,22,23,24]. Figure 1d also confirms that the oxidation of a thin film occurs not only in the AlN film, but also in the TaN film [21]. Ta-N bonding accounted for the largest percentage; however, oxidized Ta bonding is observed. The approximately same ratio of Ta-N-O bonding and Al-N-O bonding is calculated in Figure 1e,f.

**Figure 2:**
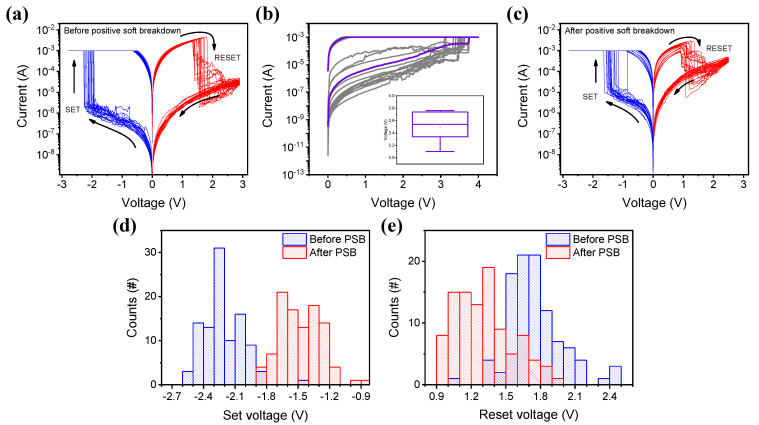
Typical I–V curves of Pt/AlN/TaN device (**a**) before positive soft breakdown (PSB). (**b**) Positive soft breakdown and the distribution of PSB voltage in inset. (**c**) Then, 100 consecutive switching curves after PSB. (**d**) Distribution of the set voltage before and after PSB. (**e**) The reset voltage distribution.

To characterize and understand the resistive switching mechanism of the Pt/AlN/TaN device, I–V characteristics were investigated before and after the standard of PSB that induces the stress on the device. As shown in Figure 2a, the device does not need an electroforming process for a typical bipolar resistive switching. Inferred from the above XPS data, the plentiful oxygen vacancies inside the AlON layer induced a free-forming property [22]. The current abruptly changes from the high resistance state (HRS) to the low resistance state (LRS) during the set process in the negative bias, with a compliance of 1 mA. The compliance prevents the devices from hard breakdown, which leads to device failure. Conversely, reset operation occurs in the positive bias, and the current decreases when a positive voltage of 3 V is applied to the Pt electrode. Moreover, there was a switching behavior after PSB when the same compliance and voltage of higher than 3 V are used in Figure 2b. The box chart inserted in Figure 2b indicates the distribution of the voltage inducing the current increase. The I–V characteristics in Figure 2c show the same bipolar resistive switching as before PSB; however, a clear difference between before and after PSB is also identified. Figure 2d illustrates the distribution of switching voltage as a histogram and shows the overall voltage required for switching decreases after PSB. The maximum voltage for the switching transition increases from −2.60 V to −1.88 V and from +2.48 V to +1.94 V, respectively. Since the required voltage for operation is reduced, the PSB induces the explosive growth of conductive filaments and makes the device operate well even at low voltages [23]. In addition, it might suggest that there is a variation between the front and rear switching mechanisms.

**Figure 3:**
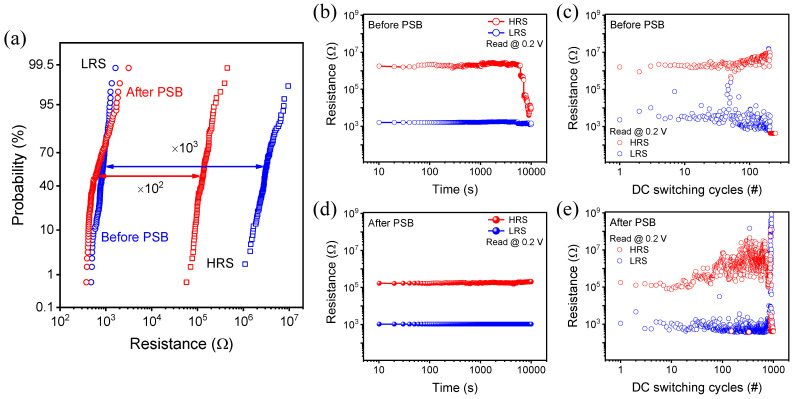
(**a**) Cumulative probability of each state resistance (blue line indicates before PSB, red line indicates after PSB). (**b**) Retention and (**c**) endurance for 250 cycles using a read voltage of 0.2 V before PSB. (**d**) Retention and (**e**) endurance for 1000 cycles tested after PSB.

Further analyzing the DC characteristics, the resistance values at the read voltage of 0.2 V were gathered and expressed as a cumulative probability in Figure 3a. Devices that had experienced PSB or not were grouped, and divided into each figure in the LRS and HRS. While the average value of LRS rarely changes from 852 Ω to 773 Ω due to the compliance control, HRS average values decrease significantly from 3.32 MΩ to 144 kΩ. The decrease in HRS is the effect of PSB, which is different from the typical RRAM that causes hard breakdown. The retention for storing information was tested in Figure 3b,d. When proceeding with the test for 10^4^ s at room temperature, HRS that had not experienced the PSB could not maintain a constant value. The instability of HRS further confirmed in the endurance test that the switching failure occurred just as it reached 200 DC cycles. Compared to this, the tests in Figure 3c,e show improved memory performance, with exquisite retention over 10^4^ s and superior endurance up to 900 DC cycles including few switching failure points. Consequently, the PSB reduces the on/off ratio; however, it strengthens the information-storing ability and rewriting ability.

**Figure 4:**
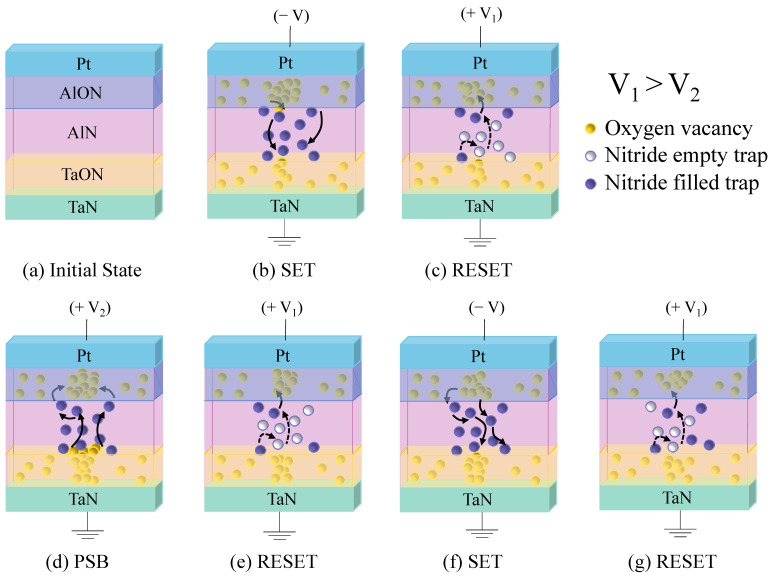
Schematics of switching process: (**a**) initial state, (**b**) set, and (**c**) reset process. Schematics of switching process after positive soft breakdown: (**d**) PSB, (**e**) first reset after PSB, (**f**) set, and (**g**) reset process.

Figure 4 displays the schematic model of non-volatile memory based on the electrical measurement data and XPS results. The initial state before the negative switching is depicted in Figure 4a. Oxygen vacancies are scattered across AlON and TaON layers formed by factors such as natural oxidation and process limitations. The paper with the AlO_x_N_y_-based RRAM previously reported that resistive switching can be conducted in low oxygen concentrations [21,22,24]. The conductive mechanism on the basis of oxygen migration can be explained, and our device also contains irresistible oxygen inside the switching medium by the XPS spectrum [24]. When the oxynitride layer is deposited on the nitride thin film, it may be seen that the oxynitride layer contributes to the formation of the conductive filament and the capture of the nitride traps [25]. Thus, when the negative voltage is applied to the Pt electrode, electrons are injected into the AlN layer passing through, and the oxygen vacancies formed in AlON and TaON go to fill the empty nitride traps [25]. Those filled traps eject electrons with a positive voltage, and the conductive filament is cut off and converted from LRS to HRS, as shown in Figure 4c. Despite the following resistive switching, PSB exceeding the general reset voltage induces the current increase until it reaches the compliance level. As can be observed from the XPS data in Figure 1e, the bonding energy is large in the order of Ta-O-N to Al-O-N [19,26]. Therefore, it is easier to break the Ta-O-N bonding under a low electric field and reconstitute the thicker conductive filament with oxygen vacancies in the TaON layer, as shown in Figure 4d [27]. However, the principle of resistive switching remains that the filled nitride traps release electrons in the direction of the Pt electrode to maintain the reset process in Figure 4g. As a result, HRS is strengthened with the thicker filament, while similar bipolar switching occurs [28].

**Figure 5:**
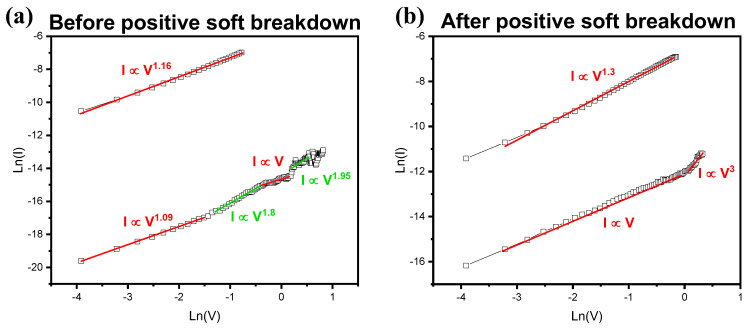
Ln (I) versus Ln (V) for (**a**) before positive soft breakdown (PSB) and (**b**) after PSB.

To accurately analyze the effects of PSB, the conduction mechanism was examined in Figure 5. The I–V curve was fitted with the plot of ln (V) versus ln (I) corresponding to the Ohmic conduction. In both Figure 5a,b, LRS approximately fitted with the slope of 1, which follows Ohmic behavior. However, HRS corresponding to space charge limited conduction (SCLC) shows different gradient changes. HRS divided into four regions in Figure 5a matches the slope change of the general SCLC mechanism. Each slop of a, b, c, and d correlates with the region of Ohmic, trap-limited SCLC, trap-filled SCLC, and trap-free SCLC [29]. On the other hand, Figure 5b is separated into only two regions of Ohmic and trap-filled SCLC. This indicates that the space charge region is very small, and that the nitride trap attached to the filaments by PSB is already filled with the injected electrons [30,31]. Analysis conducted with electrical measurements figures out the effect of PSB, and reveals the influence of oxynitride layers deposited by wrapping nitride thin film up and down similar to a sandwich in the filament conductive mechanism.

**Figure 6:**
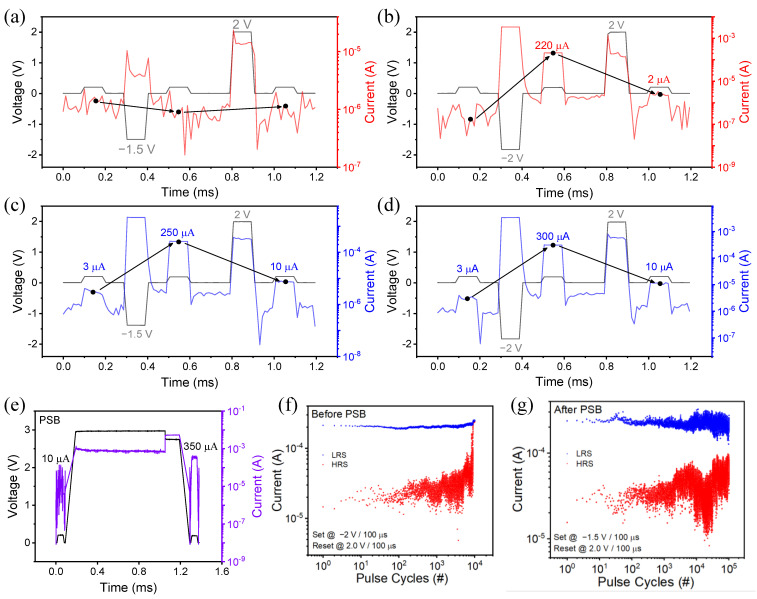
Transient characteristics of set/reset pulse inputs before PSB (**a**) set: −1.5 V/100 μs, reset: 2 V/100 μs; (**b**) set: −2 V/100 μs, reset: 2 V/100 μs and after PSB; (**c**) set: −1.5 V/100 μs, reset: 2 V/100 μs; (**d**) set: −1.5 V/100 μs, reset: 2 V/100 μs; and (**e**) PSB: 3 V/1 mA. Pulse endurance properties for (**f**) before PSB and (**g**) after PSB.

Figure 6 revealed that the PSB behavior appeared even in pulse mode. As illustrated in Figure 6a, where a set voltage −1.5 V is applied at 100 μs of pulse width in an initial state, a current is not changed; however, where −2 V is applied, the current is increased up to 220 μA; then, the current is reduced by applying a reset voltage of 2 V (Figure 6b). Figure 6c,d shows the transient characteristics after the PSB operation. It can be seen that the current increases at a voltage of −2 V or higher before PSB, whereas the current is converted from HRS to LRS even when the set pulse of −1.5 V is applied and the conversion from HRS to LRS is performed. When a positive voltage of 3 V is applied as a pulse width of 1 ms, the current falls and then rapidly increases, resulting in the formation of a conductive filament (Figure 6e). Figure 6f,g depicts a comparison of the pulse endurance properties before and after the PSB. Prior to the PSB (Figure 6f), when the set/reset pulses of −2 V/100 μs and 2 V/100 μs were applied 10,000 times, the HRS current surged at the end and the switching failed. On the other hand, after the PSB (Figure 6g), the cycle was repeated over 100,000 times with a set pulse of −1.5 V/100 μs, which can be shown to be measured similarly to the DC endurance characteristic.

**Figure 7:**
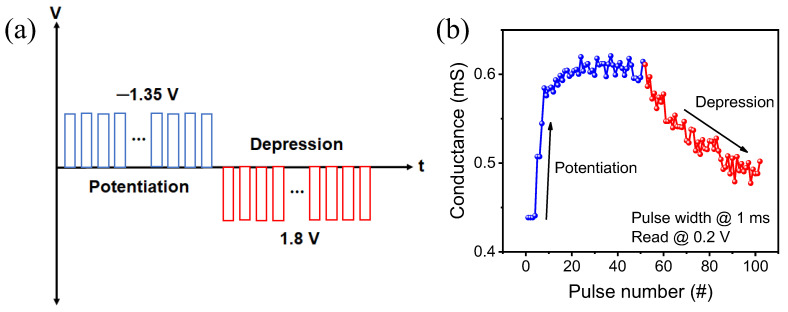
(**a**) Identical 50 pulses for potentiation and depression characteristics; and (**b**) conductance change in potentiation (blue)/depression (red).

Finally, the potentiation and depression characteristics are demonstrated in Figure 7 by applying 50 identical pulses. As seen in Figure 7a, the pulse width is fixed to 1 ms, and the potentiation and depression operations are carried out to raise and lower the conductance, respectively, to −1.35 V and 1.8 V. To measure the degree of change in conductance, the read voltage is applied between the set and reset voltages. When a set voltage is applied, the conductance changes abruptly and it gradually declines in depression, precisely as the DC I–V characteristics shown in Figure 3a,c (Figure 7b). As a result, AlN-based memristors exhibit abrupt pulse properties and have a rapid response time.

## 3. Materials and Methods

The Pt/AlN/TaN RRAM device was fabricated as follows. First, we purchased a TaN/SiO_2_/Si substrate from GEMK Inc. TaN deposition was conducted in the condition of DC reactive sputtering. A power of 250 W was used at room temperature. The substrate was split into pieces and cleaned in order with the chemicals of alcohol, isopropyl alcohol, and deionized water at the sonicator for 5 min, respectively. ALD (NCD, Lucida M300PL-O) was used for the growth of a 5 nm AlN thin film. The metal precursor of trimethylaluminum (Al(CH_3_)_3_, TMA) and the reaction gas of ammonia (NH_3_) were injected into the chamber, and the sequence proceeds as follows: TMA feeding → N_2_ gas purge → NH_3_ feeding → RF(NH_3_) feeding → N_2_ gas purge. It was repeated for 59 cycles at the 450 °C temperature stage to deposit AlN, atomic-by-atomic. The shadow mask with a circular pattern of diameter 100 μm was attached, and the e-beam evaporator (ULVAC, FF-EB20) was conducted for Pt electrode deposition. Analysis instruments of the transmission electron microscope (TEM) and the semiconductor parameter analyzer (Keithley 4200-SCS, Cleveland, OH, USA) were used to specify the memory performance and electrical properties.

## 4. Conclusions

In this paper, the electrical properties of Pt/AlN/TaN devices were investigated by the DC voltage application. Based on the PSB, a type of breakdown, the filament conductive mechanism in an AlN thin film was analyzed. XPS surface examination validated the chemical bonding and material composition of the AlN/TaN film. We succeeded in achieving improved endurance and retention through PSB. It stabilized metastable HRS and showed data retention of 10^4^ s, and endurance of 900 DC switching cycles. The switching mechanism based on the roles of oxygen vacancies and nitride traps was modeled with the schematic, and explained using linear fitting of the I–V curve. By measuring the transient characteristics and the PSB behavior in pulse mode, more current flow after PSB was confirmed as in DC mode. Finally, identical pulse schemes were used to achieve the potentiation and depression, indicating that this device can serve as a synaptic device. From this work, we reveal that the operating method can be the key point of RRAM application; thus, an optimal operating algorithm must be configured to maximize the performance of RRAM.

## Data Availability

Not applicable.
